# Somatic Overgrowth Predisposes to Seizures in Autism Spectrum Disorders

**DOI:** 10.1371/journal.pone.0075015

**Published:** 2013-09-23

**Authors:** Giulia Valvo, Sara Baldini, Francesca Brachini, Fabio Apicella, Angela Cosenza, Anna Rita Ferrari, Renzo Guerrini, Filippo Muratori, Maria Francesca Romano, Filippo M. Santorelli, Raffaella Tancredi, Federico Sicca

**Affiliations:** 1 Epilepsy, Neurophysiology and Neurogenetics Unit, IRCCS Stella Maris Foundation, Pisa, Italy; 2 Developmental Psychiatry Unit, IRCCS Stella Maris Foundation, Pisa, Italy; 3 Child Neurology Unit, A. Meyer Pediatric Hospital, University of Florence, Florence, Italy; 4 Institute of Economics, Sant'Anna School of Advanced Studies, Pisa, Italy; 5 Molecular Medicine Unit, IRCCS Stella Maris Foundation, Pisa, Italy; College of Pharmacy, University of Florida, United States of America

## Abstract

**Background:**

Comorbidity of Autism Spectrum Disorders with seizures or abnormal EEG (Autism-Epilepsy Phenotype) suggests shared pathomechanisms, and might be a starting point to identify distinct populations within the clinical complexity of the autistic spectrum. In this study, we tried to assess whether distinct subgroups, having distinctive clinical hallmarks, emerge from this comorbid condition.

**Methods:**

Two-hundred and six individuals with idiopathic Autism Spectrum Disorders were subgrouped into three experimental classes depending on the presence of seizures and EEG abnormalities. Neurobehavioral, electroclinical and auxological parameters were investigated to identify differences among groups and features which increase the risk of seizures. Our statistical analyses used ANOVA, post-hoc multiple comparisons, and the Chi-squared test to analyze continuous and categorical variables. A correspondence analysis was also used to decompose significant Chi-squared and reduce variables dimensions.

**Results:**

The high percentage of children with seizures (28.2% of our whole cohort) and EEG abnormalities (64.1%) confirmed that the prevalence of epilepsy in Autism Spectrum Disorders exceeds that of the general population. Seizures were associated with severe intellectual disability, and not with autism severity. Interestingly, tall stature (without macrocephaly) was significantly associated with EEG abnormalities or later onset seizures. However, isolated macrocephaly was equally distributed among groups or associated with early onset seizures when accompanied by tall stature.

**Conclusions:**

Tall stature seems to be a phenotypic “biomarker” of susceptibility to EEG abnormalities or late epilepsy in Autism Spectrum Disorders and, when concurring with macrocephaly, predisposes to early onset seizures. Growth pattern might act as an endophenotypic marker in Autism-Epilepsy comorbidity, delineating distinct pathophysiological subtypes and addressing personalized diagnostic work-up and therapeutic approaches.

## Introduction

Autism spectrum disorders (ASD) are characterized by a wide range of dysfunctions in communicative and social ability, and by repetitive, restricted, and stereotyped interests and behaviors. However, clinical presentation of ASD is extremely heterogeneous, reflecting different degrees of severity and perhaps multiple pathogenetic backgrounds. Children with ASD have a higher risk of developing seizures (5–46%) [1] when compared to the general population (0.5–1%), and up to one in three subjects with ASD displays electroencephalographic (EEG) abnormalities without seizures [2]. These findings suggest that, in some patients, ASD, epilepsy and EEG abnormalities may share common genetic causes or, possibly, pathophysiological mechanisms [3], [4]–[6], and that this comorbidity - termed Autism-Epilepsy Phenotype (AEP) [3], [7], [8] - deserves further investigation. The complexity of AEP, however, which possibly reflects multifaceted pathomechanisms causing this comorbid condition, makes it difficult to ascertain the actual relationships [4], [9], [10].

Over the past few years, a number of studies have assessed the comorbidity of ASD and epilepsy [1] by analyzing features such as time of seizures onset (infantile versus pubertal) [3], [11], [12], presence of developmental regression [13]–[17], macrocephaly [18], or motor problems [19] without reaching a unifying mechanism or conclusive results. A recent meta-analysis has suggested that intellectual disability and female gender represent significant risk factors for the development of seizures in ASD [20]. Moreover, a latent class cluster analysis has defined a distinct subgroup of ASD showing epilepsy, early diagnosis, and distinctive neurobehavioral features [9]. Nonetheless, the link between ASD and epilepsy remains largely elusive, probably because of the heterogeneity of samples which included, in most studies, children with “non idiopathic” ASD or symptomatic seizures, or even lacking a formal diagnosis of epilepsy.

In this study, we attempted a more detailed analysis of the phenotypic features of children with ASD, with and without epilepsy/EEG abnormalities, to pinpoint distinctive characteristics associated with this comorbid condition. Measures of cognitive and socio-behavioral symptoms, as well as electro-clinical features and auxological parameters, alone or in combination, were investigated to explore specific phenotypic traits associated with risk of seizures (or EEG abnormalities) in ASD, and to foster the clustering of affected individuals.

## Materials and Methods

### Ethics Statement

This study was approved by the Research Ethics Committee of the IRCCS Fondazione Stella Maris, Pisa (Italy). All patients or their parents signed an informed consent prior to the assessment, and agreed for their medical data to be used anonymously in future research.

### Participants

We collected and reviewed the clinical data of 206 individuals with ASD who underwent EEG recordings in consecutive hospitalizations between January 2010 and September 2012 in a third-level center for ASD diagnosis. All subjects had received a clinical, neurobehavioral assessment and EEG evaluation as part of a routine diagnostic work-up. Individuals with a past history of seizures or abnormal EEG also received expert advice for epilepsy.

The sample consisted of 174 boys (M) (84.5%) and 32 girls (F) (15.5%) (M:F = 5:1), aged 2.2 to 20.8 years (yrs) (mean age 7.1; standard deviation (SD) 3.8). The sample was divided into 3 subgroups: 1. ASD with a history of seizures (ASD-seizures); 2. ASD with EEG abnormalities, but without seizures (ASD-EEG); 3. ASD without seizures and with normal EEG (ASD “simplex”) (Table 1). We defined as “EEG abnormalities” focal or diffuse spikes, sharp waves, and/or spike and wave complexes, and/or focal slowing.

**Table 1:** Characteristics of total sample and experimental groups.

	Total sample	Experimental Groups					
		ASD-seizures	ASD-EEG	ASD “simplex”	Effect size	Test	p
**Sample size**	206	58 (28.2%)	76 (36.9%)	72 (35%)	-	χ^2^ = 2.602	0.270
**Age assessment (yrs)** *							
mean±sd	7.1±3.8	9.4±4.9	6.6±3	5.7±2.6	η = 0.398	F = 19.093	<0.001*
range	2.2–20.8	2.2–20.8	2.4–17.5	2.2–15.1			
C.I. (95%)	6.5–7.5	8.1–10.7	5.8–7.3	5.1–6.3			
**Gender**							
Boys	174 (84.5%)	51 (29.3%)	58 (33.3%)	65 (37.4%)	Φc = 0.174	χ^2^ = 6.232	0.044
Girls	32 (15.5%)	7 (21.9%)	18 (56.2%)	7 (21.9%)			
**ASD Diagnosis**							
Autism	73 (35.4%)	22 (30.1%)	24 (32.9%)	27 (37%)	Φc = 0.096	χ^2^ = 3.767	0.430
PDD-NOS	130 (63.1%)	34 (26.2%)	52 (40%)	44 (33.8%)			
Asperger	3 (1.5%)	2 (66.7%)	0 (0%)	1 (33.3%)			
EEG abnormalities**							
Yes	132 (64.1%)	56 (42.4%)	76 (57.6%)	-	Fisher’s Exact Test		<0.001**
No	74 (35.9%)	2 (2.7%)	-	72 (97.3%)			

* one-way ANOVA, post-hoc Bonferroni method, ASD-seizures > ASD-EEG, p<0.001; ASD-seizures > ASD “simplex”, p<0.001; ASD-EEG vs ASD “simplex”, p>0.010; **EEG abnormalities are significantly associated with the presence of seizures (56/58 children with seizures vs 76/148 without seizures).

**Φc = ** Cramers’ phi coefficient, **η = **eta, **χ^2^ = **the Pearson chi-squared test, **F = ** F of Fisher, one-way ANOVA.

All clinical and neurophysiologic data were collected on a dedicated database. Patients with non-idiopathic autism and\or symptomatic epilepsy, due to congenital or acquired cerebral lesions or known genetic syndromes, were excluded. Brain MRI was performed in 132/206 patients: almost all of the cases in the ASD-seizures group (53/58; 91.4%), and about half of the children in the remaining two groups (46/76 (60.5%) in the ASD-EEG and 33/72 (45.8%) in the ASD “simplex” group). High-resolution karyotype and Fragile-X testing were performed in 192/206 (93.2%) and 190/206 (92.2%) patients, respectively.

### Methods

The diagnosis of ASD was performed according to the criteria of the Diagnostic and Statistical Manual of Mental Disorders, Fourth Edition, Text Revision (DSM-IV TR) for Pervasive Developmental Disorders, and confirmed in 154/206 (74.8%) children with the gold standard Autism Diagnostic Observation Schedule- Generic (ADOS-G) administered by a psychologist (certified for clinical and research practice).

Video-EEG-polygraphic recordings while awake and asleep were digitally acquired, positioning electrodes on the scalp according to the 10–20 system, and visually inspected by two independent investigators. When EEG evaluations were discordant, a third opinion by a clinical expert was requested. Seizures and epilepsy phenotypes were classified according to the International League Against Epilepsy (ILAE) classifications (1981, 1989) and the subsequent Report of the ILAE Commission on Classification and Terminology (2005–2009) [21].

Auxological parameters were obtained as part of the standardized clinical examination and, when possible, retrieved by reviewing previous clinical data from primary-care pediatricians. Height (H) and weight (W) were measured by calibrated scales. Head circumference (HC) was obtained by placing a plastic, non-stretchable tape measure over the maximum occipital-frontal circumference. For reference, height measurements were converted to z-scores for age (standard deviation score, SDS) using the “SIEDP Growth Calculator” based on the Cacciari and colleagues growth charts for the Italian population from 2 to 20 yrs [22]. The values of W and HC were plotted on standard growth charts [22], [23]. Weight was not included in the statistical analyses because this parameter could be affected by aberrant eating behaviors, and therefore deemed to be less reliable and difficult to correlate with other features.

Cognitive development was assessed using the following standardized tools: Griffiths Mental Development Scale Extended-Revised (GMDS-ER), Leiter International Performance Scale-Revised (LIPS-R), Wechsler Preschool and Primary School Intelligence Scale-III (WPPSI), and Wechsler Intelligence Scale for Children-III (WISC-III). When standardized evaluation was not applicable, the cognitive level was assessed through clinical observation. We considered three categories for statistical analyses, based on the IQ score (when available) and the clinician’s judgment: normal-to-borderline level, mild-to-moderate delay, and severe delay. Language development was assessed through clinical observation and classified as absent, delayed, and normal.

Parents were asked to fill the Child Behavior Checklist (CBCL 1½-5 or CBCL 6-18), a parent-report questionnaire measuring child behavioral problems. The following features were also annotated, at first examination and during the time of clinical observation, and labeled as present or absent: developmental regression, sleep problems, regulation disorders of sensory processing (hypersensitive, hyposensitive, sensory stimulation-seeking), frustration intolerance, self-injurious behavior or aggressiveness and stereotypes. Whenever possible, family history for epilepsy, febrile seizures, ASD, anxiety, mood disorders, psychotic disorders, cognitive and language delay was also investigated up to the 4th degree of kinship and carefully annotated.

### Statistical Analysis

We adopted the IBM© SPSS software version 16 for our statistical analysis. For continuous variables, we performed t-test, analyses of variance (ANOVA) and post-hoc multiple comparisons using the Bonferroni correction or Dunnet test in accordance with the Levene test on variance homogeneity. The option “exclude cases analysis by analysis” was chosen to manage missing data. To analyze categorical variables, we used the Chi-squared test and the correspondence analysis (CA) to decompose the significant Chi-squared and reduce variables dimensions. CA is a multivariate technique specifically designed to capture non linear associations between variables, when a “model-free” approach rather than an a priori assumption on the possible type of association is chosen. Its graphical display shows how levels from two or more categorical variables cluster together, allowing an overview of the salient relationships among them. Statistically significant data are indicated by asterisks in the tables. Significance was set at p≤0.010.

## Results

The three experimental groups were not significantly different with respect to sample size, gender, and ASD diagnosis (Table 1). We found a mild association between female gender and ASD-EEG group which was, however, below the cut-off for statistical significance (p = 0.044). EEG abnormalities were found in 132/206 subjects (64.1%), including almost all of the children with seizures (56/58) and about half (76/148) of those without seizures (Fisher’s Exact test, p<0.001; Table 1). Seizures were reported in 58/206 patients (28.2% of the whole sample), and in 56/132 (42.4%) children with EEG abnormalities. The age at time of clinical evaluation was significantly higher (p< 0.001) in the ASD-seizures group compared to the other two experimental groups (Table 1), probably due to the presence of individuals with adolescence-onset epilepsy in our sample. Onset of seizures in ASD, indeed, has a bimodal distribution, with one peak occurring before the age of 5 years, and a later onset after age 10 [3], [7], [8].

### Family data and neurobehavioral features

A family history for psychiatric diseases (ASD, anxiety, mood disorders, psychosis) or cognitive and language delay was equally observed in the three experimental groups (Table S1 in Supporting Information S1), whereas epilepsy and febrile seizures were more common (showing a trend to significance) in relatives of children belonging to the ASD-seizures group (Table 2).

**Table 2:** Family data, regressive onset, cognitive and language development.

	Total sample	Experimental Groups					
		ASD-seizures	ASD-EEG	ASD “simplex”	Effect size	Test	p
**Family History Epilepsy**							
Yes	41 (20.4%)	18 (43.9%)	9 (22%)	14 (34.1%)	Φc = 0.207	χ2 = 8.582	0.014
No	160 (79.6%)	37 (23.1%)	67 (41.9%)	56 (35%)			
**Family History Febrile Seizures**							
Yes	21 (10.4%)	11 (52.4%)	5 (23.8%)	5 (23.8%)	Φc = 0.192	χ2 = 7.397	0.025
No	180 (89.6%)	44 (24.4%)	71 (39.4%)	65 (36.1%)			
**Regressive onset**							
Yes	82 (41.4%)	27 (32.9%)	34 (41.5%)	21 (25.6%)	Φc = 0.173	χ2 = 5.896	0.052
No	116 (58.6%)	28 (24.1%)	39 (33.6%)	49 (42.2%)			
**Level of Cognitive Development** *							
Normal to Borderline	84 (44.2%)	15 (17.9%)	32 (38.1%)	37 (44%)	Φc = 0.187	χ2 = 13.347	**0.010** *
Mild to Moderate Delay	68 (35.8%)	21 (30.9%)	22 (32.4%)	25 (36.8%)			
Severe Delay	38 (20%)	18 (47.4%)	13 (34.2%)	7 (18.4%)			
**Level of Language Development**							
Normal	42 (20.4%)	13 (31%)	17 (40.5%)	12 (28.5%)	Φc = 0.072	χ2 = 2.121	0.714
Delayed	107 (51.9%)	27 (25.2%)	38 (35.5%)	42 (39.3%)			
Absent	57 (27.7%)	18 (31.6%)	21 (36.8%)	18 (31.6%)			

**Figure 1:**
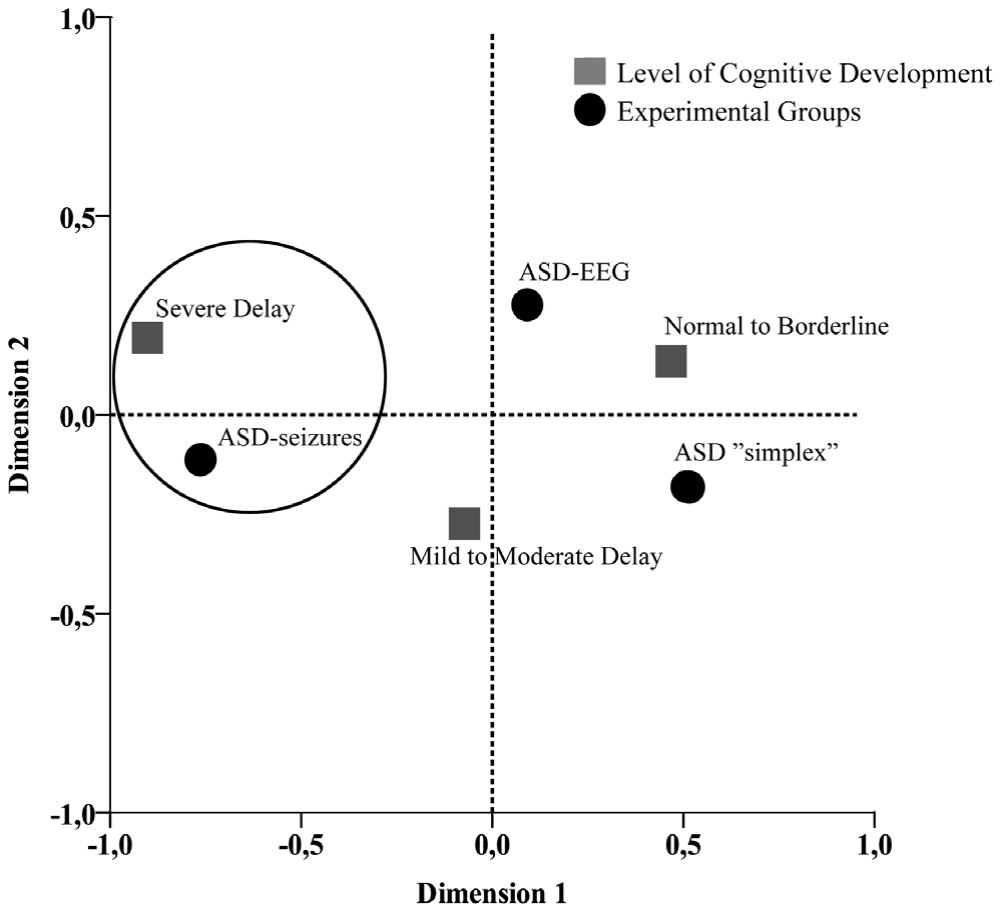
Biplot for cross-tabulation of Level of Cognitive Development by Experimental Groups. Correspondence analysis at two-dimensional solution showed that the ASD-seizures group is relatively associated with severe cognitive delay (closed circle).

* Severe cognitive delay is associated with the ASD-seizures group (see Figure 1, and CA in Table S4 in Supporting Information S1).

**Φc = ** Cramers’ phi coefficient, **χ^2^ = **the Pearson chi-squared test.

Clinical and behavioral features, assessed by the ADOS-G and CBCL scores, showed no significant differences between groups (Table S2 in Supporting Information S1). Behavioral features recorded in the clinical interviews by checking the presence/absence of sleep disorders, frustration intolerance, self/hetero injurious behavior, regulation disorders of sensory processing, and stereotyped behaviors did not differ between groups (Table S3 in Supporting Information S1).

### Regressive onset, cognitive and language development

There were no significant differences between groups with respect to the onset (regressive versus non-regressive) of ASD (Table 2).

Standardized cognitive evaluation was obtained in 155/206 individuals, whereas 35 individuals were only evaluated through expert clinical observations. Cognitive information was not available in 16 individuals. Chi-squared and CA analyses revealed that severe delay was statistically associated (p = 0.010) with the ASD-seizures group on dimension 1 (Table 2 and Figure 1; for details see Table S4 in Supporting Information S1). No significant differences were found with respect to gender. Assessment of language development revealed no significant differences across groups (Table 2).

### Auxological parameters

**Figure 2:**
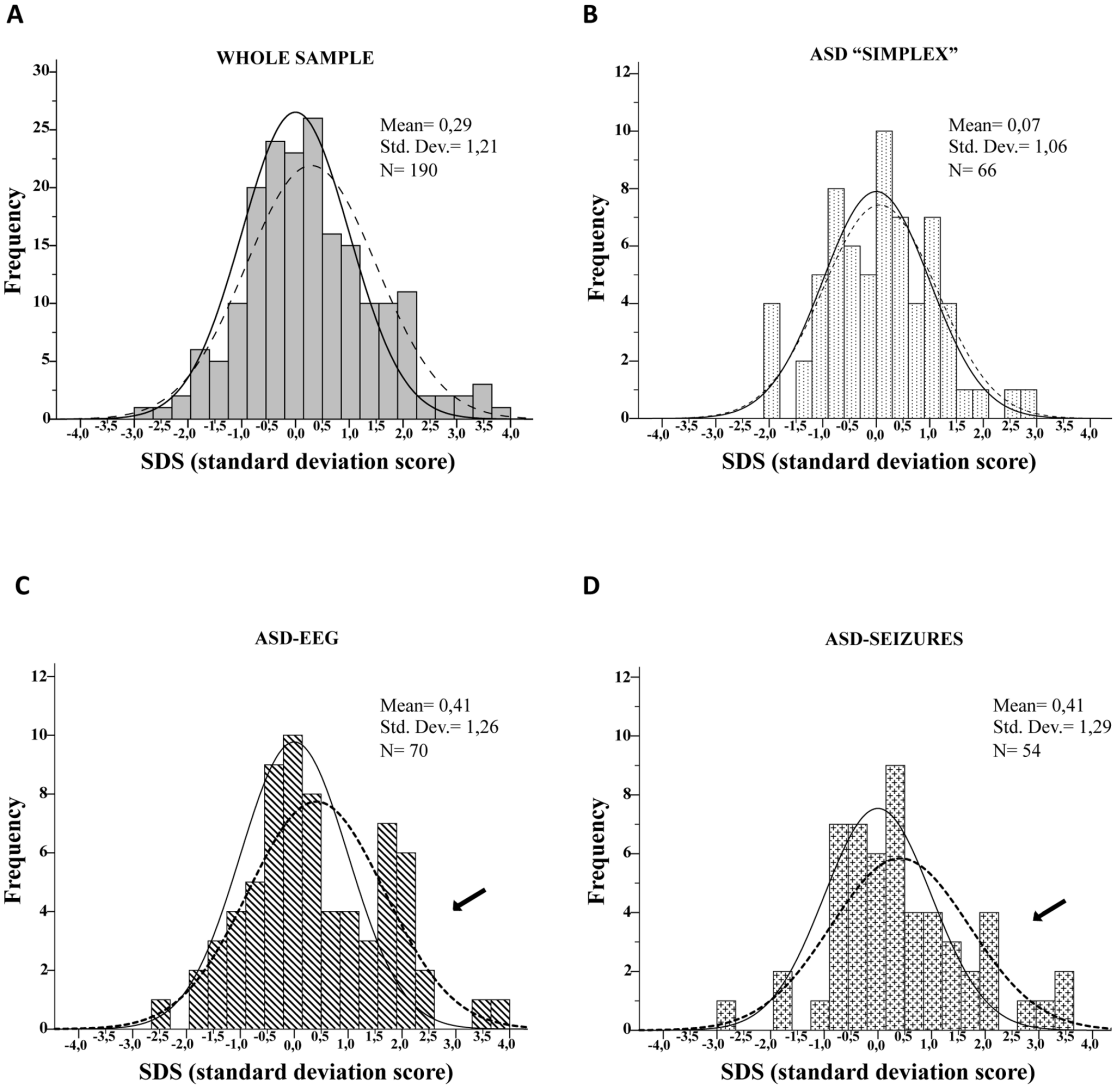
Distribution of Z-scores for height (Zh). **A.** The histogram of Zh of whole sample shows a significant rightward shift (dashed curve) compared to the normative distribution (closed curve). **B.** The Zh distribution of ASD “simplex” group shows no difference with respect to normative distribution. **C.** The Zh distribution of ASD-EEG group shows a significant rightward shift (dashed curve) compared to the normative distribution (closed curve). **D.** The Zh distribution of ASD-seizures group shows a trend towards a rightward shift (dashed curve) compared to the normative distribution (closed curve). Arrows in C and D indicate a clearly higher rate of tall individuals over 1.5 SDS.

We obtained complete auxological data in 187/206 individuals. The age (in years) at time of auxological measures ranged from 2.0 to 20.7, with a mean age of 7.1 (SD 3.7) for H, 6.9 (3.6) for W and 6.6 (3.6) for HC. The distribution of Z-scores for height (Z_h_) in the whole sample showed a normal shape (Kolmogorov-Smirnov, p = 0.036; Shapiro, p = 0.101), with significant rightward shift with respect to the normative distribution (mean: 0.29; SD: 1.21; t = 3.32, df = 189, p = 0.001; Figure 2A). By separately testing the Z_h_ distributions of the three experimental groups, we found that ASD “simplex” did not differ from the normative distribution (mean: 0.07; SD: 1.06; t = 0.508, df = 65, p = 0.613, Figure 2B). Conversely, there was a significant rightward shifted Z_h_ distribution (mean: 0.41; SD: 1.26; t = 2.727, df = 69, p = 0.008, Figure 2C) in the ASD-EEG group and a trend towards a rightward shift (mean: 0.41; SD: 1.29; t = 2.355, df = 53, p = 0.022, Figure 2D) in the ASD-seizures group because of the presence of a high rate of tall individuals in the upper boundary of the distributions. In particular, 10/54 (18.5%) children in the ASD-seizures group, and 19/70 (27.1%) in the ASD-EEG group had height measures over 1.5 SDS (93^rd^ percentile), which is considerably more than 7% expected in the normative population (Figure 2C, 2D). On the other hand, only 4/66 (6.1%) children in the ASD simplex group were over the 93^rd^ percentile which is similar to normative data in the healthy population. To further explore this finding, we considered height over 1.5 SDS as a categorical variable (tall stature) and found that it was strongly unrelated to the ASD “simplex” group, whereas it was positively associated with the ASD-EEG group, and weakly associated with the ASD-seizures group (p = 0.005; Table 3, and Table S5 in Supporting Information S1).

**Table 3:** Auxological parameters.

	Total sample	Experimental Groups					
		ASD-seizures	ASD-EEG	ASD “simplex”	Effect size	Test	p
**Tall Stature (>1.5 SDS)** *							
Yes	33 (17.4%)	10 (30.3%)	19 (57.6%)	4 (12.1%)	Φc = 0.236	χ2 = 10.590	**0.005** *
No	157 (82.6%)	44 (28%)	51 (32.5%)	62 (39.5%)			
**Macrocephaly**							
Yes	53 (27.9%)	18 (34%)	21 (39.6%)	14 (26.4%)	Φc = 0.120	χ2 = 2.756	0.252
No	137 (72.1%)	35 (25.5%)	49 (35.8%)	53 (38.7%)			
Auxological Category**							
AUX1	119 (63.6%)	33 (27.7%)	36 (30.3%)	50 (42%)	Φc = 0.333	χ2 = 20.680	0.002**
AUX2	35 (18.7%)	10 (28.6%)	14 (40%)	11 (31.4%)			
AUX3	17 (9.1%)	8 (47.1%)	6 (35.3%)	3 (17.6%)			
AUX4	16 (8.6%)	2 (12.5%)	13 (81.2%)	1 (6.2%)			

**AUX1**: Normal head circumference and height; **AUX2**: Isolated Macrocephaly; **AUX3**: Macrocephaly and Tall Stature; **AUX4**: Isolated Tall Stature.

* Tall Stature is strongly unrelated to the ASD “simplex” group, and is associated with the ASD-EEG group (see CA in Table S5 in Supporting Information S1). **Isolated Tall Stature (AUX4) is associated with EEG abnormalities; concurrence of Tall Stature and Macrocephaly (AUX3) is associated to seizures; Isolated Macrocephaly (AUX 2) is equally distributed among groups (see CA in Table S6 in Supporting Information S1).

**Φc = ** Cramers’ phi coefficient, **χ2 = **the Pearson chi-squared test.

Macrocephaly (HC over the 97^th^ percentile) was found in 53/190 (27.9%) patients in the whole sample; however its occurrence was not significantly different in the three groups (Table 3).

**Figure 3:**
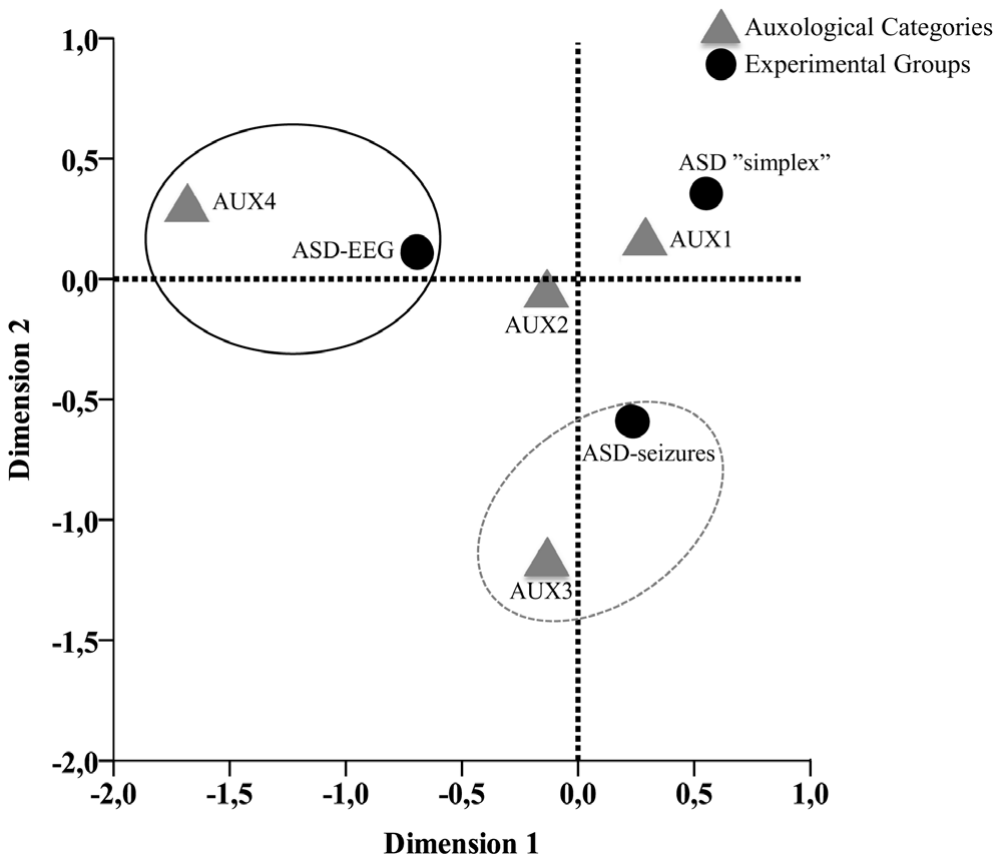
Biplot for Cross-Tabulation of Auxological Categories by Experimental Groups. Correspondence analysis at two-dimensional solution showed that ASD-EEG group is relatively associated with Isolated Tall Stature (AUX4, closed circle), and ASD-seizures group is relatively associated with combined macrocephaly and tall stature (AUX3, dashed circle).

In order to better understand the relation between HC and H, auxological features were decomposed and combined in a single “auxological variable” for each individual defining four major categories: Aux1.Normal HC and H; Aux2.Macrocephaly combined with normal stature (Isolated Macrocephaly); Aux3.Macrocephaly with tall stature; Aux4.Tall stature combined with normal HC (Isolated Tall Stature). The distribution of each category in the total sample and in the three experimental groups is summarized in Table 3. CA on this “auxological variable” vs. the experimental groups showed a two-dimensional solution (see Table 3 and Figure 3). On dimension 1, the largest contribution to inertia (80%) indicated a significant association between isolated tall stature and EEG abnormalities (without seizures). On dimension 2, concurrence of tall stature and macrocephaly was significantly associated with seizures. Isolated macrocephaly was equally distributed among groups (Figure 3, and Table S6 in Supporting Information S1).

Mean ages at latest clinical assessment were 7.6 (SD 4.0) years (range 2.9–15.7) in the Aux3 category (tall stature and macrocephaly) and 7.0 (2.9) years (3.6–14.3) in the Aux4 category (tall stature and normal HC) (t = 0.542, df  = 31, p = 0.592) whereas mean ages at seizure onset were 5.0 (3.0) years (0.7–9.2) in Aux3 and 12.5 (2.5) years (10.8–14.3) in Aux4 (t =  3.258, df  = 8, p = 0.012). The clear trend to significance suggested that children with isolated tall stature had a delayed onset of seizures when compared to tall, macrocephalic children.

Auxological categories did not differ with respect to all the other variables mentioned in the Methods Section.

## Discussion

In our cohort, rates of seizures (28.2%) and EEG abnormalities (64.1%) are in line with the literature data confirming that the prevalence of epilepsy in ASD noticeably exceeds that of the general population (0.5–1%) [8], [20]. EEG abnormalities were associated with seizures in 56/132 (42.4%) children, and severe intellectual disability was also associated with increased risk of seizures (p = 0.010), confirming previous findings [20], [24], though we did not observe the association of seizures with gender. However, the cognitive profile in our cohort was similar in boys and girls and it has been suggested that girls with ASD may have a higher risk of epilepsy because of a more severe degree of intellectual disability [20] (D. Cohen, personal communication, December 17, 2012). The trend to a significant association between female gender and ASD-EEG group (p = 0.044), however, suggests that this possible relationship should be further investigated in larger samples. Interestingly, we also found a higher than expected occurrence of epilepsy and/or febrile seizures in relatives of the ASD-seizures group, suggesting that a family history for seizures represents a risk factor for developing epilepsy in ASD. Like other features of AEP the susceptibility to seizures may be a single part, inherited from less penetrant or non-affected parents, of a more complex disorder and contributes, through interaction with multiple genetic and environmental factors, to the overt phenotype. Alternatively, the phenotype may be caused by *de novo* mutations in developmental genes [25], [26]. ADOS scores did not differ between groups in our sample suggesting that seizures (or EEG abnormalities) do not affect *per se* the core behavioral, communicative and social features of ASD.

The most relevant finding of the present work was that seizures and EEG abnormalities were significantly related to somatic growth patterns. The rates of children with stature over 1.5 SDS (17.4%) and with macrocephaly (27,9%) in our sample were considerably higher than expected in the general population, suggesting that overgrowth in ASD may involve the whole body and not be limited to the brain [27]. There is conflicting literature about macrocephaly in ASD, in part due to different methodological approaches, such as the time point of head measurements, and size of the samples investigated. While some authors do not find significant differences between ASD and healthy children [28], [29], others report rates of macrocephaly ranging from 17 to 53% [30]–[32], and in line with our data. Recent findings indicate that cranial overgrowth in ASD appears in the first year of life, suggesting that a variety of mechanisms accelerating brain growth in early development could predispose to autism [30], [31], [33]–[36]. However, specific processes responsible for early brain overgrowth in ASD have not been identified. Even less clear are the issues related to the prevalence and significance of other growth parameters such as W and H in ASD. Recent studies have focused on the different prevalence of tall stature in ASD compared to healthy infants, emphasizing an overall disturbance of somatic growth regulation, rather than a specific dysregulation of neural development [27], [33], [37]. This observation also suggests that generalized overgrowth might represent a “biomarker” associated with one or more homogeneous clinical and pathophysiological subtypes of ASD [33]. However, the relationship between somatic growth and seizures (or EEG abnormalities) in ASD has not been fully investigated and deserves further research. We found that tall stature (without macrocephaly) was significantly associated with EEG abnormalities without seizures, whereas concurrence of tall stature and macrocephaly was significantly associated with the overt autism-seizures phenotype. Only 2/16 children with isolated tall stature exhibited seizures, at age 10.8 and 14.3, respectively. Onset of seizures in ASD has a bimodal distribution, with one peak occurring before the age of 5, and a later onset after age 10 [3], [7], [8]. Indeed, the presence of individuals with adolescence-onset epilepsy in our sample may explain the higher mean age of the ASD-seizures patients compared to the other two experimental groups. Although patients in the Aux3 and Aux4 groups were assessed at a comparable age, children with macrocephaly and tall stature (Aux3) had an earlier onset of seizures (age 5 at the first peak for seizures), compared to children with isolated tall stature (Aux4) who displayed later seizures at a mean age of 12.5; the statistical analysis of the age at seizure onset in the two groups showed a strong trend to significance (p = 0.012). We may infer, indeed, that most children in the Aux4 group, at the time of assessment, might not have displayed seizures as yet, because too young in relation to mean age for onset of seizures in that group, indicating the need to further confirm this finding through a longitudinal follow-up. Therefore, while the combination of macrocephaly and tall stature seems to point out the possibility of early seizures in ASD, isolated tall stature might signal a “red flag” for monitoring EEG abnormalities, or the risk of manifesting seizures in early teens or adolescence, although the small number of patients having seizures in the Aux4 group (only two subjects) limits that conclusion.

Isolated macrocephaly was equally distributed among our groups, suggesting that brain overgrowth is not a factor directly affecting the risk of seizures in ASD. This finding could be interpreted under two possible scenarios. It is possible that the different patterns of exaggerated growth (isolated tall stature, isolated macrocephaly, tall stature plus macrocephaly) define distinct latent genetic and pathophysiological variables (or disorders) with diverse clinical and developmental features. A more attractive alternative is that isolated tall stature and global overgrowth are equally pieces of the same puzzling biological process. This could result from the interwork of largely unknown factors affecting both skeletal and brain growth and development. As paroxysmal EEG and seizures are considered part of a spectrum, which in different degrees denotes a similar neurobiological disorder underlain by abnormal neuronal excitability, it is tempting to hypothesize that the concurrence of tall stature and macrocephaly may represent the extreme effect of polygenic factors that affect not only the core behaviors of ASD but also susceptibility and precocity in manifesting seizures.

In conclusion, this study suggests that tall stature is a possible “biomarker” of susceptibility to EEG abnormalities or late epilepsy in ASD and, when concurring with macrocephaly, predisposes to early onset seizures. We propose that the identification of endophenotypic markers might help disclose distinct pathophysiological and genetic mechanisms in ASD and assist in clinical dissection, diagnostic work-up, and rational follow-up in AEP.

## Supporting Information

Supporting Information S1 **Supporting Tables. Table S1:** Family Data. **Table S2:** ADOS-G and CBCL Scores. **Table S3:** Neurobehavioral Features. **Table S4:** Correspondence Analysis of Level of Cognitive Development vs. Experimental Groups. Severe delay was statistically associated with the ASD-seizures group. **Table S5**: Correspondence Analysis of Tall Stature vs. Experimental Groups. Tall stature was unrelated to the ASD “simplex” group, whereas it was positively associated with the ASD-EEG group, and weakly associated with the ASD-seizures group. **Table S6:** Correspondence Analysis of Auxological Variable vs. Experimental Groups. Isolated tall stature was significantly associated with EEG abnormalities, whereas concurrence of tall stature and macrocephaly was associated with seizures. Isolated macrocephaly was equally distributed among groups.
(DOC)
