# Supplementary material for: Somatic Overgrowth Predisposes to Seizures in Autism Spectrum Disorders
Source: PLoS One. 2013 Sep 23;8(9):e75015. doi: 10.1371/journal.pone.0075015 (PMC3781047; doi:10.1371/journal.pone.0075015)
Supplement: Supporting Information S1 — Supporting Tables. Table S1: Family Data. Table S2: ADOS-G and CBCL Scores. Table S3: Neurobehavioral Features. Table S4: Correspondence Analysis of Level of Cognitive Development vs. Experimental Groups. Severe delay was statistically associated with the ASD-seizures group. Table S5: Correspondence Analysis of Tall Stature vs. Experimental Groups. Tall stature was unrelated to the ASD “simplex” group, whereas it was positively associated with the ASD-EEG group, and weakly associated with the ASD-seizures group. Table S6: Correspondence Analysis of Auxological Variable vs. Experimental Groups. Isolated tall stature was significantly associated with EEG abnormalities, whereas concurrence of tall stature and macrocephaly was associated with seizures. Isolated macrocephaly was equally distributed among groups. (DOC) [file pone.0075015.s001.doc]

**Table S1**

**Family Data**

|  | **Total sample** | **Experimental Groups** | | | | | |
| --- | --- | --- | --- | --- | --- | --- | --- |
|  |  | **ASD-seizures** | **ASD-EEG** | **ASD “simplex”** | **Effect size** | **Test** | **p** |
| **Autism Spectrum Disorders** |  |  |  |  |  |  |  |
| Yes | 29 (14.6%) | 9 (31%) | 10 (34.5%) | 10 (34.5%) | Φc=0.022 | χ^2^=0.094 | 0.954 |
| No | 169 (85.4%) | 48 (28.4%) | 62 (36.7%) | 59 (34.9%) |  |  |  |
| **Anxiety Disorder** |  |  |  |  |  |  |  |
| Yes | 33 (16.7%) | 8 (24.2%) | 9 (27.3%) | 16 (48.5%) | Φc=0.129 | χ^2^=3.297 | 0.192 |
| No | 165 (83.3%) | 49 (29.7%) | 63 (38.2%) | 53 (32.1%) |  |  |  |
| **Mood Disorder** |  |  |  |  |  |  |  |
| Yes | 54 (27.3%) | 15 (27.8%) | 20 (37%) | 19 (35.2%) | Φc=0.014 | χ^2^=0.038 | 0.981 |
| No | 144 (72.7%) | 42 (29.2%) | 52 (36.1%) | 50 (34.7%) |  |  |  |
| **Psychosis** |  |  |  |  |  |  |  |
| Yes | 9 (4.5%) | 3 (33.3%) | 3 (33.3%) | 3 (33.3%) | Φc=0.022 | χ^2^=0.098 | 0.952 |
| No | 189 (95.5%) | 54 (28.6%) | 69 (36.5%) | 66 (34.9%) |  |  |  |
| **Cognitive and Language Delay** |  |  |  |  |  |  |  |
| Yes | 72 (36%) | 19 (26.4%) | 27 (37.5%) | 26 (37.7%) | Φc=0.037 | χ^2^=0.268 | 0.875 |
| No | 128 (64%) | 38 (29.7%) | 47 (36.7%) | 43 (33.6%) |  |  |  |

**Φc=** Cramers’ phi coefficient, **χ^2^=** the Pearson chi-squared test

**Table S2**

**ADOS-G and CBCL Scores**

|  | **Experimental Groups** | | | | | |
| --- | --- | --- | --- | --- | --- | --- |
|  | **ASD-seizures** | **ASD-EEG** | **ASD “simplex”** | **Effect size** | **Test** | **p** |
| **ADOS-G** |  |  |  |  |  |  |
| A | 4.88 (±1.86) | 4.67 (±1.91) | 5.05 (±1.70) | η=0.094 | F=0.577 | 0.56 |
| B | 9.00 (±2.85) | 8.57 (±2.93) | 8.19 (±2.83) | η=0.106 | F=0.745 | 0.47 |
| C | 2.31 (±1.37) | 2.18 (±1.40) | 2.03 (±1.31) | η=0.039 | F=0.218 | 0.80 |
| D | 2.85 (±1.14) | 2.52 (±1.75) | 2.47 (±1.77) | η=0.082 | F=0.244 | 0.78 |
| E | 1.25 (±1.89) | 0.18 (±0.4) | 0.20 (±0.63) | η=0.086 | F=2.597 | 0.09 |
| **Child Behavior Checklist Mother§** |  |  |  |  |  |  |
| internalizing | 61.39 (±11.6) | 62.23 (±8.3) | 61.93 (±9.5) | η=0.031 | F=0.060 | 0.94 |
| externalizing | 56.7 (±13.2) | 57.11 (±8.8) | 58.02 (±10.1) | η=0.053 | F=0.173 | 0.84 |
| total | 60.43 (±14.1) | 60.87 (±10.1) | 61.37 (±9.9) | η=0.033 | F=0.060 | 0.94 |
| anxiety/depression | 61.17 (±10.6) | 60.04 (±7.8) | 59.82 (±9.8) | η=0.053 | F=0.177 | 0.83 |
| withdraw/depression | 61.73 (±10.6) | 59.40 (±8.2) | 58.77 (±8.18) | η=0.124 | F=0.962 | 0.38 |
| somatic complaints | 57.65 (±7.6) | 55.45 (±6.6) | 57.91 (±8.3) | η=0.152 | F=1.457 | 0.23 |
| social problems | 68.91 (±10.2) | 70.62 (±10.1) | 68.58 (±9.2) | η=0.097 | F=0.593 | 0.55 |
| thought problems | 59.26 (±7.4) | 57.57 (±8.1) | 58.4 (±8.7) | η=0.074 | F=0.338 | 0.71 |
| attention problems | 64.09 (±9.7) | 64.85 (±9.8) | 62.95 (±7.99) | η=0.096 | F=0.579 | 0.56 |
| rule-breaking behavior | 58.22 (±10.2) | 57.52 (±7.4) | 58.18 (±8.6) | η=0.037 | F=0.085 | 0.91 |
| aggressive behavior | 55.67 (±4.2) | 63.10 (±5.2) | 62.30 (±10.8) | η=0.376 | F=1.899 | 0.17 |
| **Child Behavior Checklist Father§** |  |  |  |  |  |  |
| internalizing | 60.58 (±10.6) | 62.43 (±7.5) | 60.28 (±9.0) | η=0.114 | F=0.684 | 0.50 |
| externalizing | 54.16 (±14.0) | 57.78 (±8.7) | 56.00 (±9.2) | η=0.130 | F=0.879 | 0.41 |
| total | 58.53 (±13.5) | 61.90 (±9.1) | 59.40 (±9.4) | η=0.135 | F=0.957 | 0.38 |
| anxiety/depression | 61.63 (±10.6) | 59.53 (±7.6) | 58.85 (±8.9) | η=0.114 | F=0.680 | 0.50 |
| withdraw/depression | 61.68 (±12.7) | 59.28 (±9.0) | 58.11 (±7.6) | η=0.139 | F=1.014 | 0.36 |
| somatic complaints | 54.63 (±5.3) | 55.90 (±6.6) | 55.04 (±5.8) | η=0.082 | F=0.351 | 0.70 |
| social problems | 67.63 (±10.4) | 70.68 (±8.9) | 68.06 (±9.4) | η=0.142 | F=1.058 | 0.35 |
| thought problems | 58.53 (±10.0) | 58.50 (±8.4) | 56.89 (±6.8) | η=0.100 | F=0.516 | 0.59 |
| attention problems | 62.21 (±8.2) | 65.10 (±9.1) | 62.70 (±7.7) | η=0.148 | F=1.158 | 0.31 |
| rule-breaking behavior | 57.37 (±11.5) | 57.80 (±7.2) | 55.91 (±6.9) | η=0.110 | F=0.635 | 0.53 |
| aggressive behavior | 55.17 (±2.9) | 64.33 (±5.0) | 63.33 (±5.7) | η=0.667 | F=7.201 | <0.01 |

**A**: Language & Communication; **B**: Reciprocal Social Interaction; **C**: Play; **D**: Stereotyped Behaviors & Restricted Interests; **E**: Other Abnormal Behaviors.

**§** the scores have been obtained from the two CBCL’s versions: 1½-5 and 6-18; **η**=eta, **F**= F of Fisher, one-way ANOVA

**Table S3**

**Neurobehavioral Features**

|  | **Total Sample** | **Experimental Groups** | | | | | |
| --- | --- | --- | --- | --- | --- | --- | --- |
|  |  | **ASD-seizures** | **ASD-EEG** | **ASD “simplex”** | **Effect size** | **Test** | **p** |
| **Sleep Disorders** |  |  |  |  |  |  |  |
| Yes | 53 (26.6%) | 16 (30.2%) | 20 (37.7%) | 17 (32.1%) | Φc=0.043 | χ^2^=0.373 | 0.830 |
| No | 146 (73.4%) | 39 (26.7%) | 54 (37%) | 53 (36.3%) |  |  |  |
| **Frustrations Intolerance** |  |  |  |  |  |  |  |
| Yes | 157 (77.7%) | 38 (24.2%) | 58 (36.9%) | 61 (38.9%) | Φc=0.171 | χ^2^=5.907 | 0.052 |
| No | 45 (22.3%) | 18 (40%) | 17 (37.8%) | 10 (22.2%) |  |  |  |
| **Self/Hetero-Injurious Behavior** |  |  |  |  |  |  |  |
| Yes | 118 (58.4%) | 33 (28%) | 41 (34.7%) | 44 (37.3%) | Φc=0.063 | χ^2^=0.810 | 0.667 |
| No | 84 (41.5%) | 23 (27.4%) | 34 (40.5%) | 27 (32.1%) |  |  |  |
| **Regulation Disorders of Sensory Processing** |  |  |  |  |  |  |  |
| Yes | 139 (69.5%) | 38 (27.3%) | 50 (36%) | 51 (36.7%) | Φc=0.048 | χ^2^=0.465 | 0.792 |
| No | 61 (30.5%) | 15 (24.6%) | 25 (41%) | 21 (34.4%) |  |  |  |
| **Stereotyped Behavior** |  |  |  |  |  |  |  |
| Yes | 178 (88.1%) | 47 (26.4%) | 68 (38.2%) | 63 (35.4%) | Φc=0.05 | χ^2^=0.532 | 0.767 |
| No | 24 (11.9%) | 8 (33.3%) | 8 (33.3%) | 8 (33.3%) |  |  |  |

**Φc**= Cramers’ phi coefficient, **χ**^2^ = the Pearson chi-squared test.

**Table S4**

**Correspondence Analysis of Level of Cognitive Development vs. Experimental Groups**

| **Summary** | | | | | | | | | | | | | | | |
| --- | --- | --- | --- | --- | --- | --- | --- | --- | --- | --- | --- | --- | --- | --- | --- |
| **Dimension** | **Inertia** | | **Chi Square** | | **Sig.** | | **Proportion of Inertia** | | | | **Confidence Singular Value** | | | | |
|  |  |  |  |  |  |  | Accounted for | | Cumulative | | Standard Deviation | | | Correlation | |
|  |  |  |  |  |  |  |  |  |  |  |  |  |  | 2 | |
| **1** | 0.068 | |  | |  | | 0.974 | | 0.974 | | 0.070 | | | -0.152 | |
| **2** | 0.002 | |  | |  | | 0.026 | | 1.000 | | 0.071 | | |  | |
| **Total** | 0.070 | | 13.347 | | 0.010 | | 1.000 | | 1.000 | |  | | |  | |
| **Overview Column Points** | | | | | | | | | | | | | | | |
| **Experimental Groups** | | **Score in Dimension** | | | | **Inertia** | | **Contribution** | | | | | | | |
|  | | **1** | | **2** | |  | | Of Point to Inertia of Dimension | | | | Of Dimension to Inertia of Point | | | |
|  |  |  |  |  |  |  |  | **1** | | **2** | | **1** | **2** | | **Total** |
| **ASD-seizures** | | -0.763 | | -0.112 | | 0.043 | | 0.632 | | 0.084 | | 0.997 | 0.003 | | 1.000 |
| **ASD-EEG** | | 0.092 | | 0.276 | | 0.002 | | 0.011 | | 0.636 | | 0.408 | 0.592 | | 1.000 |
| **ASD “simplex”** | | 0.507 | | -0.181 | | 0.025 | | 0.357 | | 0.280 | | 0.980 | 0.020 | | 1.000 |
| **Active Total** | |  | |  | | 0.070 | | 1.000 | | 1.000 | |  |  | |  |
| **Overview Row Points** | | | | | | | | | | | | | | | |
| **Level of Cognitive Development** | | **Score in Dimension** | | | | **Inertia** | | **Contribution** | | | | | | | |
|  | | **1** | | **2** | |  | | Of Point to Inertia of Dimension | | | | Of Dimension to Inertia of Point | | | |
|  |  |  |  |  |  |  |  | **1** | | **2** | | **1** | **2** | | **Total** |
| **Normal to Borderline** | | 0.468 | | 0.134 | | 0.026 | | 0.370 | | 0.188 | | 0.987 | 0.013 | | 1.000 |
| **Mild to Moderate Delay** | | -0.073 | | -0.274 | | 0.002 | | 0.007 | | 0.635 | | 0.306 | 0.694 | | 1.000 |
| **Severe Delay** | | -0.903 | | 0.194 | | 0.043 | | 0.623 | | 0.177 | | 0.993 | 0.007 | | 1.000 |
| **Active Total** | |  | |  | | 0.070 | | 1.000 | | 1.000 | |  |  | |  |

CA (two dimensions): **1° Dimension** (97% of total inertia): **in column (Experimental Groups)** ASD-seizures group has a higher contribution to inertia (63%, negative sign) than ASD-EEG (1%, positive sign) and ASD “simplex” (36%, positive sign); **in row (Level of Cognitive Development)** Severe Delay has a higher contribution to inertia (62%, negative sign) than Mild to Moderate Delay (1%, negative sign) and Normal to Borderline (37%, positive sign). The **2° Dimension** has a 3% of total inertia.

**Table S5**

Correspondence Analysis of Tall Stature vs. Experimental Groups

| **Summary** | | | | | | | | | | | |
| --- | --- | --- | --- | --- | --- | --- | --- | --- | --- | --- | --- |
| **Dimension** | **Inertia** | | **Chi Square** | | **Sig.** | | **Proportion of Inertia** | | | **Confidence Singular Value** | |
|  |  |  |  |  |  |  | Accounted for | Cumulative | | Standard Deviation | |
| **1** | 0.056 | |  | |  | | 1.000 | 1.000 | | 0.061 | |
| **Total** | 0.056 | | 10.590 | | 0.005 | | 1.000 | 1.000 | |  | |
| **Overview Column Points** | | | | | | | | | | | |
| **Experimental Groups** | | **Score in Dimension** | | **Inertia** | | **Contribution** | | | | | |
|  | |  | |  | | Of Point to Inertia of Dimension | | | Of Dimension to Inertia of Point | | |
|  |  | **1** | |  |  | **1** | | | **1** | | **Total** |
| **ASD-seizures** | | -0.062 | | 0.000 | | 0.005 | | | 1.000 | | 1.000 |
| **ASD-EEG** | | -0.531 | | 0.025 | | 0.440 | | | 1.000 | | 1.000 |
| **ASD “simplex”** | | 0.614 | | 0.031 | | 0.555 | | | 1.000 | | 1.000 |
| **Active Total** | |  | | 0.056 | | 1.000 | | |  | |  |
| **Overview Row Points** | | | | | | | | | | | |
| **Tall Stature** | | **Score in Dimension** | | **Inertia** | | **Contribution** | | | | | |
|  | |  | |  | | Of Point to Inertia of Dimension | | | Of Dimension to Inertia of Point | | |
|  |  | **1** | |  |  | **1** | | | **1** | | **Total** |
| **No** | | 0.223 | | 0.010 | | 0.174 | | | 1.000 | | 1.000 |
| **Yes** | | -1,060 | | 0.046 | | 0.826 | | | 1.000 | | 1.000 |
| **Active Total** | |  | | 0.056 | | 1.000 | | |  | |  |

CA (one dimension): **in column (Experimental Groups)** total inertia is explained by ASD “simplex” for 55.5% (positive sign), ASD-EEG for 44% (negative sign) and ASD-seizures for 0.5 % (negative sign); **in row** (Tall Stature) total inertia is explained by the presence of tall stature for 83% (negative sign) and by absence of tall stature for 17% (positive sign).

**Table S6**

Correspondence Analysis of Auxological Variable vs. Experimental Groups

| **Summary** | | | | | | | | | | | | | | | | | | | | |
| --- | --- | --- | --- | --- | --- | --- | --- | --- | --- | --- | --- | --- | --- | --- | --- | --- | --- | --- | --- | --- |
| **Dimension** | **Inertia** | | | **Chi Square** | | | **Sig.** | | **Proportion of Inertia** | | | | | | **Confidence Singular Value** | | | | | |
|  |  |  |  |  |  |  |  |  | Accounted for | | | | Cumulative | | Standard Deviation | | | | Correlation | |
|  |  |  |  |  |  |  |  |  |  |  |  |  |  |  |  |  |  |  | 2 | |
| **1** | 0.089 | | |  | | |  | | 0.804 | | | | 0.804 | | 0.066 | | | | 0.047 | |
| **2** | 0.022 | | |  | | |  | | 0.196 | | | | 1.000 | | 0.076 | | | |  | |
| **Total** | 0.111 | | | 20.680 | | | 0.002 | | 1.000 | | | | 1.000 | |  | | | |  | |
| **Overview Column Points** | | | | | | | | | | | | | | | | | | | | |
| **Experimental Groups** | | | **Score in Dimension** | | | | | **Inertia** | | | **Contribution** | | | | | | | | | |
|  | | | **1** | | **2** | | |  | | | Of Point to Inertia of Dimension | | | Of Dimension to Inertia of Point | | | | | | |
|  |  |  |  |  |  |  |  |  |  |  | **1** | **2** | | **1** | | | **2** | **Total** | | |
| **ASD-seizures** | | | 0.229 | | -0.589 | | | 0.019 | | | 0.050 | 0.667 | | 0.234 | | | 0.766 | 1.000 | | |
| **ASD-EEG** | | | -0.694 | | 0.117 | | | 0.054 | | | 0.597 | 0.034 | | 0.986 | | | 0.014 | 1.000 | | |
| **ASD “simplex”** | | | 0.551 | | 0.356 | | | 0.038 | | | 0.353 | 0.299 | | 0.829 | | | 0.171 | 1.000 | | |
| **Active Total** | | |  | |  | | | 0.111 | | | 1.000 | 1.000 | |  | | |  |  | | |
| **Overview Row Points** | | | | | | | | | | | | | | | | | | | | |
| **Tall Stature** | | **Score in Dimension** | | | | **Inertia** | | | | **Contribution** | | | | | | | | | | |
|  | |  | | | |  | | | | Of Point to Inertia of Dimension | | | | | | Of Dimension to Inertia of Point | | | | |
|  |  | **1** | | | |  |  |  |  | **1** | | | | | | **1** | | | | **Total** |
| **AUX1** | | 0.284 | | | | 0.147 | | | | 0.017 | | | | | | 0.172 | | | | 0.093 |
| **AUX2** | | -0.132 | | | | -0.065 | | | | 0.001 | | | | | | 0.011 | | | | 0.005 |
| **AUX3** | | -0.135 | | | | -1.174 | | | | 0.019 | | | | | | 0.006 | | | | 0.850 |
| **AUX4** | | -1.681 | | | | 0.296 | | | | 0.073 | | | | | | 0.811 | | | | 0.051 |
| **Active Total** | |  | | | |  | | | | 0.111 | | | | | | 1.000 | | | | 1.000 |

CA (two dimensions). **1° Dimension** (80% of total inertia):  **in column (Experimental Groups)** ASD-EEG has the highest contribution to inertia (60%, negative sign), and ASD simplex has an inertia of 35% (positive sign); **in row (Auxological Categories)** AUX4 has the highest contribution to inertia (81%, negative sign), AUX1 has an inertia of 17% (positive sign). **2° Dimension** (20% of total inertia): **in column (Experimental Groups)** ASD-seizures has the highest contribution to inertia (67%, negative sign); ASD “simplex” and ASD EEG contribute to inertia respectively for the 30% and 3% (both positive sign); **in row (Auxological Categories)** AUX3 has the highest contribution to inertia (85%, negative sign); AUX1 and AUX4 have a contribution to inertia respectively for the 9% and 5% (both positive sign).
